# Calculated parenteral initial treatment of bacterial infections: Introduction and antibiotics

**DOI:** 10.3205/id000063

**Published:** 2020-03-26

**Authors:** Klaus-Friedrich Bodmann, Michael Kresken, Béatrice Grabein, Pascal M. Dohmen, Michael Wilke

**Affiliations:** 1 Klinik für Internistische Intensiv- und Notfallmedizin und Klinische Infektiologie, Klinikum Barnim GmbH, Werner Forßmann Krankenhaus, Eberswalde, Germany; 2 Antiinfectives Intelligence GmbH, Campus Hochschule Bonn-Rhein-Sieg, Rheinbach, Germany; 3 Rheinische Fachhochschule Köln gGmbH, Cologne, Germany; 4 Stabsstelle Klinische Mikrobiologie und Krankenhaushygiene, Klinikum der Universität München, Munich, Germany; 5 Klinik und Poliklinik für Herzchirurgie, Universitätsmedizin Rostock, Germany; 6 inspiring-health Dr. Wilke GmbH, Munich, Germany

## Abstract

This is the first chapter of the guideline “Calculated initial parenteral treatment of bacterial infections in adults – update 2018” in the 2^nd^ updated version. The German guideline by the Paul-Ehrlich-Gesellschaft für Chemotherapie e.V. (PEG) has been translated to address an international audience.

This guideline is a revision of the recommendations published in 2010, taking into account recent substances and studies. As with previous revisions, the current situation of pathogen resistance and the results of new clinical trials are considered. The results are the present recommendations for parenteral calculated initial therapy of bacterial infections in adults. If several treatment options are mentioned, they are not always equivalent in their spectrum of microbiological activity. Therapeutic alternatives offer the opportunity to consider pathogen epidemiology, to avoid antibiotic intolerances or to escalate or de-escalate treatment in a manner suited to the situation. This article describes the different therapy options.

## Zusammenfassung

Dies ist das erste Kapitel der von der Paul-Ehrlich-Gesellschaft für Chemotherapie e.V. (PEG) herausgegebenen S2k Leitlinie „Kalkulierte parenterale Initialtherapie bakterieller Erkrankungen bei Erwachsenen – Update 2018“ in der 2. aktualisierten Fassung.

Bei der vorliegenden Leitlinie handelt sich um die Überarbeitung der 2010 erschienenen Empfehlungen unter Berücksichtigung neuerer Substanzen und Studien. Wie bei früheren Überarbeitungen werden hierbei die aktuelle Resistenzsituation der Erreger und die Ergebnisse neuer klinischer Studien berücksichtigt. Das Ergebnis sind die vorliegenden Empfehlungen zur parenteralen kalkulierten Initialtherapie bakterieller Infektionen beim Erwachsenen. Werden mehrere Therapieoptionen genannt, sind sie in ihrem mikrobiologischen Wirkungsspektrum nicht immer gleichwertig. Therapiealternativen bieten die Möglichkeit, die Erregerepidemiologie zu berücksichtigen, Antibiotika-Unverträglichkeiten zu umgehen oder situationsadaptiert eine Therapie zu eskalieren oder zu deeskalieren. Der vorliegende Beitrag beschreibt die verschiedenen Therapiemöglichkeiten.

## Introduction

This guideline is a revision of the recommendations published in 2010 [1], taking into account recent substances and studies. As with previous revisions, the current situation of pathogen resistance and the results of new clinical trials are considered; and the substances are summarized in tabular form.

The therapy recommendations are shown with ratings The recommendation ratings shown in Table 1 (Tab. 1) apply.

The results are the present recommendations for parenteral calculated initial therapy of bacterial infections in adults. If several treatment options are mentioned, they are not always equivalent in their spectrum of microbiological activity. Therapeutic alternatives offer the opportunity to consider pathogen epidemiology, to avoid antibiotic intolerances or to escalate or de-escalate treatment in a manner suited to the situation. Thus the attending physician can better adapt their treatment decision to the risk profile of the individual patient. The PEG recommendations focus on the initial treatment of bacterial infections. As part of the establishment of strategies for securing rational antibiotic treatment – known in the English-speaking world as Antibiotic Stewardship (ABS) – the guideline-oriented choice of initial treatment plays a key role. It is one of the ABS core strategies and part of the quality assurance of ABS measures. Wrong initial treatment has negative clinical and economic effects.

The updated recommendations are therefore in line with the requirements for ABS in Germany.

## Evaluation of the approved indications for each antibiotic

Due to different approval conditions as part of the development of the Medicinal Products Act, many older antibiotics have a much wider range of approved indications than substances that were approved by the Federal Institute for Drugs and Medical Devices (BfArM) or European Medicines Agency (EMA) during the last 15–20 years. Because of the significant increase in demands on clinical development and the associated costs over this period, newer substances are often approved for only one or two indications. However, they are also used outside their areas of authorized use (off-label use) for severe or multidrug-resistant infections. 

With regard to the legal aspects of off-label use, there is a decision of the Federal Social Court of 19 March 2002 (B 1 KR 37/00 R) according to which use outside the officially approved indications are reimbursed by statutory health insurances, if

- they are being used to treat serious illnesses,
- no other treatment is available and
- based on the data, there is a reasonable chance of successful treatment.

The problems and open questions for medical practice have been set out in a statement in the Federal Health Bulletin.

Every doctor has to make their treatment decision together with the individual patient in question. The doctor will choose the treatment which is best supported by the available evidence. However, they must check whether the result of their decision-making is actually transferable to the individual patient for whom they must select treatment (integration with internal evidence). Particularly when treating infections with parenteral antibiotics, the use of different antibiotic groups to reduce selection pressure is imperative due to problems with resistant strains in intensive care units and in the hemato-oncological field, so that off-label use of microbiologically active substances in certain situations is justified, e.g. in the treatment of infections in critically ill patients or infections by pathogens that have acquired resistance to the approved antibiotics.

## Characterization of antibiotics

### Beta-lactams

Beta-lactams have a bactericidal effect and show time-dependent death kinetics. For this reason, the duration of the drug level above the minimum inhibitory concentration (T>MIC) is the most important parameter for the effectiveness of beta-lactam antibiotics.

### Penicillins

The classification of parenteral penicillins into groups is based on their structure as benzylpenicillin, aminopenicillins, acylaminopenicillins and isoxazolylpenicillins. Associated with these structural characteristics penicillins behave very differently towards pathogens compared to beta-lactamases. The post-antibiotic effect, if present, is short-lived. Information on the application of penicillins can be found in chapter 3 [2].

The pharmacokinetic properties of penicillins do not show great variability one from another. The distribution is primarily extracellular, the relative volume of distribution is 0.2–0.4 l/kg body weight. The cerebrospinal fluid penetrability of penicillins is adequate when using sufficient dosages for inflamed meninges. The plasma half-lives are 1–2 hours in patients with healthy kidneys, elimination is usually unchanged via the kidneys. Plasma protein binding is very different and can reach values of >90% in isoxazolylpenicillins. Depending on the group, the antibacterial spectrum of penicillins is narrow to very broad and the most important selection criterion for clinical use.

### Benzylpenicillin (penicillin G)

The antibacterial spectrum of penicillin G includes most streptococci, pneumococci, meningococci, spirochetes and some anaerobic pathogens such as clostridia and *Actinomyces*-species. Benzylpenicillin is effective against staphylococci in only a few cases due to the production of beta-lactamases or modified binding proteins. The approval of penicillin G allows use in almost all systemic and local infections, regardless of the location of the infection, if the infection is caused by penicillin-susceptible pathogens. Since the antibacterial spectrum is very narrow, severe infections initially should not be treated in monotherapy prior to pathogen detection. However, penicillin G is considered as the drug of choice for erysipelas and mono-infection by streptococci and pneumococci because of the favorable tissue penetration, very good tolerability and low resistance rates in Germany (data on the current resistance situation in Germany can be found in chapter 2 [3]). Patients from certain other countries (e.g. Spain) must be expected to have significantly higher rates of resistant pneumococci.

In its slow-release form, benzylpenicillin is an organic alkali salt with low solubility for intra-muscular injection. Plasma concentrations are low and peak concentrations are reached after considerable delay. Indications for slow-release penicillin include prophylaxis for preventing recurrent rheumatic fever and erysipelas and treatment of primary syphilis (Lues I).

### Isoxazolylpenicillins: flucloxacillin, oxacillin

They have a narrow antibacterial spectrum in the Gram-positive range and work well on staphylococci, including penicillinase-producing strains. These penicillin derivatives are ineffective against methicillin-resistant staphylococci. Compared with other Gram-positive pathogens, they are less active than benzylpenicillin. Therefore, they should only be used for targeted treatment of infections caused by methicillin-susceptible staphylococci.

Compared to the other penicillins, isoxazolylpenicillins show a high plasma protein binding of more than 90%.

### Aminopenicillins: ampicillin, ampicillin/sulbactam, amoxicillin/clavulanic acid

The antibacterial spectrum of aminopenicillins includes Gram-positive as well as some Gram-negative pathogens. The activity against streptococci, including the pneumococci, is good and, compared to penicillin G, even stronger against *Enterococcus* *faecalis* and listeria. Its effectiveness against staphylococci and Gram-negative pathogens, especially members of Enterobacteriaceae, *Moraxella* *catarrhalis* and *Bacteroides* *fragilis*, is very limited because of increasing pathogen resistance through production of beta-lactamases. Up to 80% of the strains show a reduced susceptibility. Combination with a beta-lactamase inhibitor (BLI) can extend the spectrum of aminopenicillins to numerous beta-lactamase-producing Gram-positive and Gram-negative pathogens as well as anaerobes, so that a calculated treatment is possible. Ampicillin is approved for the treatment of acute and chronic bacterial infections with proven susceptible pathogens, regardless of the location of the infection and the severity of the disease, including endocarditis, meningitis and sepsis. It is approved for the treatment of infections of the upper and lower respiratory tract, the kidneys and efferent urinary tract, abdomen, sexual organs, skin and soft tissue and for perioperative antibiotic prophylaxis. Amoxicillin/clavulanic acid and ampicillin/sulbactam are available on the market in a fixed combination. Sulbactam is also available for free combination.

The most common adverse effects of aminopenicillins are pseudoallergic skin reactions. A morbilliform rash usually occurs 5–10 days after the start of treatment. Patients with concurrent viral infection (e.g. infectious mononucleosis) are particularly affected.

### Acylaminopenicillins: mezlocillin, piperacillin, piperacillin/tazobactam, combinations with sulbactam

The antibacterial spectrum of acylaminopenicillins includes Gram-positive and Gram-negative pathogens. Piperacillin also affects *Pseudomonas* *aeruginosa*. Due to the high rate of beta-lactamase-producing staphylococci, but also of Enterobacteriaceae and important anaerobes, the effect of acylaminopenicillins alone is often limited. Here too, the antibacterial spectrum on beta-lactamase-producing pathogens can be extended by combination with a beta-lactamase inhibitor, so that acylaminopenicillin/BLI combinations are also suitable for calculated initial treatment of severe nosocomial infections. There is a choice between fixed combination of piperacillin with tazobactam and the free combination of mezlocillin or piperacillin with sulbactam. Tazobactam is the more effective inhibitor in vitro. In terms of evidence-based antibiotic treatment, well-documented studies, practical advantages in preparation and pharmacokinetic aspects speak in favor of fixed combination (piperacillin/tazobactam), since piperacillin and sulbactam show diverging kinetics in particular in patients with kidney deficiency, while piperacillin and tazobactam are largely absorbed, distributed and excreted in parallel.

The approved field of use of acylaminopenicillins is extensive and includes systemic and local infections by susceptible pathogens (Gram-positive, Gram-negative, aerobic, anaerobic, mixed infections), ENT infections (piperacillin only), severe systemic infections, e.g. sepsis, bacterial endocarditis, meningitis, respiratory infections, intra-abdominal infections, kidney and urinary efferent urinary tract infections, gynecological infections, skin and soft tissue infections (including burns), bone and joint infections (including osteomyelitis) and perioperative prophylaxis.

### Cephalosporins

The parenteral cephalosporins are currently divided into 5 groups in Germany according to the recommendations of PEG. Until now the only representative of group 5 was cefoxitin. As the distribution of cefoxitin in Germany ceased, the vacated position was taken over by two new cephalosporins – ceftaroline and ceftobiprole – with MRSA activity (see group 5). The pharmacodynamic properties of cephalosporins are similar to those of penicillins. Pharmacokinetic parameters show considerable differences in the elimination of individual substances. Most cephalosporins are excreted unchanged via the kidneys. The average plasma half-life in patients with healthy kidneys is about 2 hours. Deviating pharmacokinetic properties are exhibited by ceftriaxone with an average half-life of about 8 hours and predominantly biliary elimination. Cephalosporins, like the penicillins, have extracellular distribution with a relative distribution volume of 0.2–0.4 l/kg body weight. Cephalosporins are generally very well tolerated. Allergic reactions are less common than with penicillins. Cross-allergies to penicillins are quite rare (<10%). Current resistance data can be found in chapter 2 [3]. In the classification of cephalosporins only the antibacterial activity of the antibiotic was considered. 

### Cephalosporins of group 1: cefazolin

Cefazolin is mainly active against staphylococci and streptococci. However for methicillin-resistant staphylococci, cefazolin, like all other cephalosporins, is ineffective, with the exception of ceftobiprole and ceftaroline (see Cephalosporins of group 5). The proportion of susceptible Enterobacteriaceae (such as *Escherichia* *coli*, *Klebsiella* spp. etc.) has decreased in recent years. Cefazolin is particularly suitable for the treatment of infections caused by methicillin-susceptible staphylococci and for perioperative prophylaxis.

### Cephalosporins of group 2: cefuroxime

Cefuroxime has an extended spectrum in the Gram-negative range compared to cefazolin, which also includes *Haemophilus* *influenzae*. In addition, it shows good activity against methicillin-susceptible staphylococci. High resistance rates must be expected in AmpC-producing Enterobacteriaceae, such as *Enterobacter* spp. and *Citrobacter* spp. as well as in *Morganella* *morganii* and *Proteus* *vulgaris*. Its approval includes infections by susceptible pathogens in a wide range of diseases, e.g. skin/soft tissue infections, bone and joint infections, respiratory infections, infections of the kidneys and the efferent urinary tract. Sequential therapy with the oral dosage form (cefuroxime axetil) is not recommended in severe infections due to low bioavailability and reduced dose compared to parenteral administration. 

### Cephalosporins of group 3

- 3a: cefotaxime, ceftriaxone
- 3b: ceftazidime (± avibactam)
- 3c: ceftolozane (only in combination with tazobactam)

Group 3 cephalosporins have a broad antibacterial spectrum with pronounced antibacterial activity against Gram-negative bacteria. However, their antibacterial spectrum is limited by the spread of Enterobacteriaceae with “extended-spectrum” beta-lactamases (ESBL), which also deactivate the group 3 cephalosporins. However, in combination with a beta-lactamase inhibitor, ESBL-producers can also be targeted (see below). Ceftriaxone is excreted 40–50% via the hepatobiliary route and exerts a relatively high resistance selection pressure on the gastrointestinal microbiome. The in vitro activity of cefotaxime and ceftriaxone against staphylococci is weaker compared to cephalosporins of groups 1 and 2, those of ceftazidime and ceftolozane are inadequate. These two cephalosporins are not suitable for the treatment of infections in which staphylococci are suspected or detected. Ceftazidime and ceftolozane, unlike cefotaxime and ceftriaxone, are also clinically ineffective against streptococci and pneumococci. Cefotaxime and ceftriaxone (group 3a) demonstrate no, whereas ceftazidime (group 3b) and ceftolozane (group 3c), show very good efficacy against *Pseudomonas*. The approved indications of the cephalosporins of groups 3a and 3b include diseases of all organ systems, provided they are caused by susceptible pathogens. The new group 3c includes the new cephalosporin ceftolozane, which is available in fixed combination with the beta-lactamase inhibitor tazobactam. 

### Ceftolozane/tazobactam

Ceftolozane/tazobactam has good antibacterial activity against *Pseudomonas* *aeruginosa*, as well as *Escherichia* *coli* and *Klebsiella* *pneumoniae*, including most ESBL-producing strains. Ceftolozane/tazobactam is ineffective against staphylococci and anaerobes (except *Bacteroides* *fragilis)* and has no activity against carbapenem-resistant bacteria producing serine carbapenemases (e.g. KPC, OXA) or metallo-beta-lactamases (e.g. VIM, NDM). The currently approved indications are complicated intra-abdominal infections, acute pyelonephritis and complicated urinary tract infections. Approval studies in the CAP and HAP indications are currently underway.

### Ceftazidime/avibactam

Avibactam, a new beta-lactamase inhibitor, inhibits Ambler class A and C beta-lactamases as well as some class D enzymes, but not class B enzymes (i.e. metallo-beta-lactamases). In fixed combination with group 3b cephalosporin ceftazidime, avibactam improves efficacy against strains of *Pseudomonas* *aeruginosa*, *Escherichia* *coli* and *Klebsiella* *pneumoniae* that produce ESBL enzymes, AmpC beta-lactamases and certain carbapenemases such as KPC or OXA-48. Ceftazidime/avibactam has been approved since July 1, 2016 for the treatment of patients with complicated intra-abdominal infections (cIAI), complicated urinary tract infections (cUTI), and nosocomial pneumonia (including VAP).

Another approved indication is the treatment of infections caused by aerobic Gram-negative pathogens with limited treatment options. The approved dose is 3x 2.5 g ceftazidime/avibactam i.v. with an infusion time of 2 hours.

### Cephalosporins of group 4: cefepime 

Cefepime shows weak staphylococcal activity comparable to cephalosporins of group 3a and efficacy against *Pseudomonas* which is comparable to ceftazidime. Cefepime is also active in vitro against pathogens that overexpress AmpC beta-lactamases (especially *Enterobacter* spp., *Citrobacter* *freundii)*, which distinguishes it from cephalosporins in group 3. However, ESBL-producing pathogens are resistant.

### Cephalosporins of group 5: ceftaroline, ceftobiprole 

The antibacterial spectrum of ceftaroline corresponds to that of the cephalosporins of group 3a. In addition, ceftaroline has efficacy against methicillin-resistant staphylococci. The approved indications are complicated skin and soft tissue infections and community-acquired pneumonia. Ceftobiprole shows activity against Gram-negative pathogens comparable to group 4 cephalosporins and is also active against methicillin-resistant staphylococci. In addition, ceftobiprole is active in vitro against some strains of *Enterococcus* *faecalis*. Current approval covers severe skin and soft tissue infections as well as nosocomial pneumonia except ventilator-associated pneumonia (VAP). Compliance with the specified infusion time of 2 hours is necessary to avoid undesired effects [4].

### Carbapenems

Carbapenems are well-tolerated beta-lactam antibiotics, which are divided into two groups based on their antibacterial spectrum. They show a very broad antibacterial spectrum in the Gram-positive and Gram-negative range, including anaerobes and ESBL-producing pathogens. In recent years carbapenemase-producing strains have been reported in nosocomial infections. Carbapenems show no or only a reduced effect against these pathogens.* Stenotrophomonas* *maltophilia* is naturally resistant to carbapenems. Similarly, carbapenems have no effect against methicillin-resistant staphylococci and against *Enterococcus* *faecium*. Group 1 includes imipenem (in combination with cilastatin) and meropenem. Cilastatin is an inhibitor of renal dehydropeptidase-I, which metabolizes imipenem. Group 2 includes ertapenem. Ertapenem, unlike group 1, has no clinical efficacy against *Pseudomonas* spp. and *Acinetobacter* spp.

Another distinguishing feature are the pharmacokinetic parameters. The distribution of carbapenems is extracellular, the relative volume of distribution is between 0.1 l/kg body weight (ertapenem) and 0.2 l/kg body weight (imipenem, meropenem). Binding to human serum proteins is >90% for ertapenem, about 20/40% for imipenem/cilastatin and about 2% for meropenem. All carbapenems are partially metabolized and preferentially eliminated renally. The half-life in patients with healthy kidneys is approximately one hour for group 1 carbapenems. Ertapenem has a longer half-life (about 4 hours) and is administered once a day. Imipenem/cilastatin and meropenem are dose-equivalent. For less susceptible pathogens and severe infections, a longer infusion time is recommended for meropenem, and the stability of imipenem/cilastatin is insufficient for prolonged infusion or continuous administration. For all carbapenems (as with all penicillins), a dose-dependent epileptogenic adverse drug reaction (ADR) is known. Such ADRs are reported most frequently with imipenem, (imipenem >ertapenem >meropenem). The substance is not suitable for the treatment of CNS infections. Meropenem is the only carbapenem approved for the treatment of meningitis.

### Monobactams: aztreonam

Aztreonam shows similar pharmacokinetic and pharmacodynamic behavior to other beta-lactams. It works exclusively against Gram-negative pathogens, including *Pseudomonas* *aeruginosa*. *Acinetobacter* spp., *Stenotrophomonas* *maltophilia* and ESBL-producing Enterobacteriaceae are resistant. On the other hand, metallo-beta-lactamase (MBL)-forming strains are susceptible. Due to the structural differences from the other beta-lactam antibiotics, cross-reactivity is unlikely. The clinical relevance of aztreonam is (still) low. It can be used as a combination partner with antibiotics that only work in the Gram-positive range. In the future, however, aztreonam could gain in importance, because the combination with avibactam, which is currently in clinical development is also effective against bacterial strains that produce certain serine carbapenemases such as KPC or OXA-48.

### Fluoroquinolones

The division of fluoroquinolones into 4 groups is according to the recommendations of PEG. Since parenterally available substances are only present in groups 2–4, only these groups are considered here. Fluoroquinolones show concentration-dependent bactericidal activity. The antibacterial spectrum is broad. The differences between the groups are indicated in the following sections. The high resistance rates of *Escherichia* *coli* and other Enterobacteriaceae significantly limit the use of fluoroquinolones in monotherapy as a calculated initial treatment, especially in nosocomial infections. There is usually cross-resistance between all fluoroquinolones. Fluoroquinolones are distributed extra- and intracellularly. They have a high relative volume of distribution of usually 2–4 l/kg body weight and penetrate well into many tissues. Protein binding is usually below 40%. Levofloxacin is eliminated almost exclusively via the kidneys, ciprofloxacin is also eliminated via the gall bladder and transintestinally. Moxifloxacin is largely eliminated by conjugation reactions. The half-life is 3–4 hours for ciprofloxacin, 7–8 hours for levofloxacin and more than 10 hours for moxifloxacin, which explains the different application frequency.

Undesirable effects occur in approximately 4–10% of treated patients, usually as a gastrointestinal disorder, CNS reaction in the form of insomnia and drowsiness or skin reaction. In February 2017, on the initiative of the BfArM, the EMA launched a procedure for antibiotics from the fluoroquinolone and quinolone group, which re-evaluates all reports of serious side effects that may lead to severe limitations and potentially permanent adverse effects. Amongst other things, it aims to answer the question whether the risk of known serious side effects has an impact on the risk-benefit balance. This is especially true regarding their use for the treatment of less severe infections such as acute bacterial sinusitis, acute exacerbation of chronic sinusitis, acute exacerbation of chronic bronchitis or uncomplicated urinary tract infections. The US Food and Drug Administration (FDA) had already adjusted the warnings and product information in 2016 to ensure fluoroquinolones are prescribed less freely for certain infections.

### Fluoroquinolones of group 2: ciprofloxacin, (ofloxacin)

Ciprofloxacin is very effective against Gram-negative enterobacteria and *Haemophilus* *influenzae*. It is effective against *Pseudomonas* *aeruginosa*, less effective against staphylococci and clinically not adequately effective against pneumococci and enterococci. The efficacy against chlamydia, legionella and mycoplasma is less pronounced than that of the group 3 and 4 fluoroquinolones. Approved indications are uncomplicated and complicated infections of the kidneys and/or the urinary tract, ENT, respiratory tract (not pneumococci), of the abdomen, genital organs, bones and joints, skin and soft tissues, sepsis and infections in neutropenic patients.

The use of ofloxacin is no longer recommended (see below).

### Fluoroquinolones of group 3: levofloxacin

Levofloxacin is the left-handed enantiomer and thus the effective portion of the racemate ofloxacin. This means levofloxacin shows double the antibacterial activity compared to ofloxacin. In addition, it can be given in higher doses than ofloxacin. Compared to ciprofloxacin it has a greater effect against Gram-positive pathogens such as staphylococci, streptococci, pneumococci as well as against legionella, chlamydia and mycoplasma. The effect against Gram-negative pathogens is comparable to that of ciprofloxacin, but slightly lower against *Pseudomonas* *aeruginosa*. 

Levofloxacin is approved for the treatment of community-acquired pneumonia, complicated urinary tract infections and skin and soft tissue infections.

### Fluoroquinolones of group 4: moxifloxacin

Structurally, moxifloxacin has a significantly greater effect compared to the fluoroquinolones of groups 2 and 3 against Gram-positive pathogens such as staphylococci and streptococci, including pneumococci. The effect against legionella, chlamydia and mycoplasma is further increased. Moxifloxacin is the only fluoroquinolone which is effective against Gram-positive and Gram-negative anaerobes. In contrast, it is not effective enough against *Pseudomonas* *aeruginosa*.

Moxifloxacin is approved for the treatment of community-acquired pneumonia, and for the treatment of complicated skin and soft tissue infections.

### Macrolides and azalides: erythromycin, clarithromycin, azithromycin

Macrolides possess good antibacterial activity against mycoplasma, legionella and chlamydia and against streptococci, including pneumococci, and *Bordetella* *pertussis*. The resistance rates of pneumococci were already over 20% but show a downward trend. Information on this can be found in chapter 2 [3]. The clinical efficacy of the macrolides against *Haemophilus* *influenzae* is adequate, if at all, only in high doses. Although the microbiological efficacy of clarithromycin and its active metabolites apart from azithromycin is higher than that of erythromycin, its clinical efficacy is also considered inadequate. Macrolides are mostly bacteriostatic but can also have a bactericidal effect at higher concentrations. The pharmacodynamic effect is time-dependent. Macrolides are distributed intra- and extracellularly. In addition to their antibacterial activity, macrolides also have an immunomodulatory effect. The pharmacokinetic parameters of macrolides depend on the dose and also, in the case of erythromycin, on the type of derivative. The half-life is less than 2.5 hours for erythromycin, between 2 and 5 hours for clarithromycin, and over 14 hours for azithromycin. Substantial differences are also shown in the distribution volumes: erythromycin ca. 0.7 l/kg body weight, clarithromycin ca. 4 l/kg body weight, azithromycin ca. 25 l/kg body weight. The macrolides are subject to pronounced metabolism via the liver and are eliminated preferably via the gall bladder. The most common side effects of macrolides are gastrointestinal disorders and raised liver enzymes. The high interaction potential of erythromycin and clarithromycin as well as the prolongation of the QTc time caused by all macrolides including azithromycin are problematic.

Approved indications are respiratory tract infections (especially by *Chlamydophila* *pneumoniae* or legionella) as well as the treatment of whooping cough, diphtheria, scarlet fever and erysipelas.

### Glycopeptides

#### Vancomycin, teicoplanin

The mechanism of action of the glycopeptides is based on the inhibition of cell wall synthesis, characterized by binding to the D-Ala-D-Ala terminus of the peptide side chain. Vancomycin and teicoplanin are exclusively active in the Gram-positive range. Their antibacterial spectrum includes staphylococci, including methicillin-resistant strains, streptococci, enterococci, including *Enterococcus* *faecium*, Corynebacteria and *Clostridium* *difficile*. Glycopeptide resistance in *Staphylococcus* *aureus* has been reported worldwide only in isolated cases; teicoplanin-resistant strains occur in the coagulase-negative staphylococci. Glycopeptides should only be used if, due to resistances or allergies, better-tolerated substances are out of the question, since they are clinically less effective than beta-lactams against susceptible pathogens. Glycopeptides are time-dependent with a slow-onset therapeutic effect. The volume of distribution of vancomycin is 0.4–0.9 l/kg body weight, that of teicoplanin is 1 l/kg body weight. The pharmacokinetic parameters are subject to very strong inter- and intra-individual fluctuations. The plasma half-life of vancomycin is usually 4–6 hours, that of teicoplanin 70–100 hours. The protein binding is also different: vancomycin 55%, teicoplanin 90%. The elimination of the glycopeptides is predominantly via the kidneys in unchanged form. Glycopeptides have a substance-dependent nephro- and ototoxic potential. Therapeutic drug monitoring (TDM) is therefore required for vancomycin. In patients with renal insufficiency alternative substances should be used. When administering vancomycin, the prescribed dilution and infusion time must be observed to prevent Red Man syndrome. The approved indications include sepsis, endocarditis, bone and joint infections, respiratory tract, skin and soft tissue, kidney and urinary tract infections.

#### Oritavancin, telavancin, dalbavancin

There is a new subgroup within the group of glycopeptide antibiotics, the so-called complex semi-synthetic lipoglycopeptides oritavancin, telavancin and dalbavancin. These derivatives have a bactericidal effect against Gram-positive cocci such as staphylococci (including methicillin-resistant strains) and enterococci (including partially vancomycin-resistant strains). The effect of the lipoglycopeptides is based not only on the inhibition of cell wall synthesis but also on the destabilization of the bacterial cytoplasmic membrane. 

Oritavancin is approved for the treatment of acute bacterial skin and soft tissue infections caused by methicillin-resistant strains (MRSA), as well as vancomycin-resistant *Staphylococcus* *aureus* (VRSA), vancomycin-intermediate *Staphylococcus* *aureus* (VISA) and heterogeneous VISA (hVISA). Compared to vancomycin, oritavancin has a 4 to 6 times greater effect against streptococci and enterococci, including vancomycin-resistant enterococci (VRE). The efficacy of oritavancin against VRE includes both VanA-type and Van-B-type strains. Furthermore, oritavancin is highly effective against *Clostridium* *difficile*, superior to that of metronidazole and vancomycin. Due to the very long half-life of 393 hours, this antibiotic can be used as a “single shot” therapy. 

Telavancin is also a vancomycin analogue approved for the treatment of hospital-acquired MRSA pneumonia. However, this derivative is not indicated for “first-line therapy” but should only be used if other treatments are not suitable or have failed. Undesirable outcomes are arrhythmogenic, nephrotoxic, presumably teratogenic and ototoxic effects. Telavancin should not be used in severe kidney disease and during pregnancy. It is also effective in VRSA, VISA, hVISA and VanB type VRE infections but not in VanA type infections. Furthermore, it shows very good activity (independent of penicillin resistance) against *Corynebacterium* spp., *Peptostreptococcus* spp. and *Clostridium* spp.

Dalbavancin is a teicoplanin analog and is approved for the treatment of complicated skin and soft tissue infections. The half-life is 187 hours. For this reason, this antibiotic must only be administered twice. A recent study shows that even a single treatment at a higher dose is sufficient [5]. The antibacterial spectrum of dalbavancin includes staphylococci (MSSA and MRSA), coagulase-negative staphylococci (CoNS), VISA and hVISA, but not VRSA. Dalbavancin is active against enterococci and vancomycin-resistant enterococci (VRE) of the VanB and VanC types but not against VanA. There is also good efficacy against penicillin-resistant *Streptococcus* *pneumoniae* (PRSP). It is also very effective against other Gram-positive aerobic and anaerobic microorganisms, e.g. *Corynebacterium* spp., *Listeria* spp. and *Bacillus* spp. and *Peptostreptococcus* spp. 

### Aminoglycosides: amikacin, gentamicin, tobramycin

These are effective in the Gram-negative range, especially against Enterobacteriaceae. Tobramycin and amikacin are more effective than gentamicin against *Pseudomonas aeruginosa*. The effect against Gram-positive pathogens is less pronounced. However, for instance against enterococci infections, they are used in combination with beta-lactam antibiotics to increase their effect.

Aminoglycosides show pronounced, rapid-onset, concentration-dependent bactericidal activity. The serum or tissue concentration should, if possible, exceed at least 10 times the minimum inhibitory concentration (MIC) of the pathogen. The post-antibiotic effect of the aminoglycosides may last for several hours, depending on the serum concentration, the combination partner and the immune status of the patient. The effect of the aminoglycosides is dependent on the pH. In an acidic and anaerobic environment they are ineffective. Aminoglycosides are distributed extracellularly and are eliminated unchanged via the kidneys. The relative distribution volume is approx. 0.25 l/kg body weight with a fluctuation range of 0.1–0.8 l/kg body weight. The plasma half-life is approximately 2 hours in patients with healthy kidneys but significantly longer times occur in patients with renal impairment. Therefore, especially in high-risk patients, creatinine clearance must be taken into account; TDM is required. Especially in combination treatment with beta-lactam antibiotics they should be given in a single administration of the total daily dose rather than the conventional 3x daily dosage to achieve the highest possible peak concentration. There are indications of a lower toxicity rate with more favorable clinical results for single daily doses. Within a 24 hour dosing interval, therapeutic target ranges are <1 mg/l talc concentrations and (extrapolated) peak concentrations of 15–20 mg/l for gentamicin and tobramycin and approximately 60 mg/l for amikacin in patients with normal renal function. Aminoglycosides are antibiotics with a pronounced oto- and nephrotoxic potential, they should be used only after strict indication. When used properly (once daily, short duration of treatment, TDM), they should be considered as antibiotics with acceptable tolerability (see chapter 4 [6]). Approved indications include severe (nosocomial) infections by Gram-negative rods, fever in neutropenia and *Pseudomonas* infections in cystic fibrosis. Aminoglycosides should never be given in monotherapy for these treatments. They are usually combined with a beta-lactam antibiotic. In combination with aminopenicillins they are used for the treatment of enterococcal endocarditis and for infections caused by listeria. As a rule, the aminoglycosides are only used for short-term treatment (3–5 days).

### Oxazolidinones: linezolid, tedizolid

The oxazolidinones only act against Gram-positive pathogens. They show good activity against Gram-positive cocci such as staphylococci (including methicillin-resistant strains) and enterococci (including vancomycin-resistant enterococci, VRE). There is a bactericidal effect against streptococci and a bacteriostatic effect against staphylococci and enterococci. The relative volume of distribution of linezolid is given as ca. 0.6 l/kg body weight, the protein binding is 30%, the half life is 5–7 hours. The elimination is mainly via the kidneys.

Linezolid is approved for the treatment of community-acquired and nosocomial pneumonia as well as complicated skin and soft tissue infections. During treatment, blood counts must be performed to detect possible thrombocytopenia. The duration of treatment should not exceed 28 days.

Tedizolid is a second-generation oxazolidinone and has 4 to 8 times higher activity in vitro against Gram-positive pathogens than linezolid. The substance is approved for the treatment of acute bacterial skin and soft tissue infections. In the approval study, tedizolid was found to have statistically less gastrointestinal adverse events and thrombocytopenia after 6 days than linezolid at 10 days with the same efficacy.

### Lincosamides: clindamycin

Clindamycin shows a predominantly bacteriostatic, time-dependent effect on staphylococci, streptococci, *Bacteroides* species, Corynebacteria and *Mycoplasma* *pneumoniae*. Because of its mechanism of action, clindamycin inhibits toxin production in staphylococci and streptococci, making it an important combination partner in infections where toxin activity is clinically prominent. The relative volume of distribution is about 0.6 l/kg body weight, the half-life is 2–3 hours. More than 80% of clindamycin is converted to active metabolites. Approved indications include the treatment of infections by clindamycin-susceptible pathogens of the bones and joints, including septic arthritis, infections in the dental, maxillary, ENT, the deep respiratory, pelvic and abdominal, skin, skin appendages and soft tissue as well as scarlet fever, sepsis and endocarditis.

### Tetracyclines: doxycycline

The antibacterial spectrum of doxycycline includes Gram-positive and Gram-negative pathogens as well as chlamydia and mycoplasma. Doxycycline is primarily bacteriostatic and shows both extracellular and intracellular antimicrobial activity. The relative volume of distribution is 0.8 l/kg body weight, the half-life is about 10–22 hours. Doxycycline is metabolized to a small extent and predominantly eliminated via the gall bladder but also via the kidneys. The approved indications for doxycycline are very general and include the treatment of infections by susceptible pathogens, preferably in the areas of ear, nose and throat, respiratory, urogenital and gastrointestinal tract, biliary tract and Lyme disease. Intravenous doxycycline is now the drug of choice, among others for the treatment of rickettsiosis, plague, brucellosis and Q fever.

### Glycylcyclines: tigecycline

Tigecycline has a broad antibacterial spectrum, which also includes multidrug-resistant Gram-positive pathogens such as MRSA and VRE as well as multi-resistant Gram-negative pathogens such as ESBL-producing Enterobacteriaceae and multi-resistant *Acinetobacter* *baumannii*. Furthermore, the antibacterial spectrum of the substance includes anaerobes and chlamydia, mycoplasma and legionella. Tigecycline is not active against *Pseudomonas* *aeruginosa*, *Proteus* spp., *Morganella* *morganii* and *Providencia* spp. The mode of action is primarily bacteriostatic. A bactericidal effect could also be shown [7], [8] against some pathogens, such as *Streptococcus* *pneumoniae* and *Haemophilus* *influenzae*. The volume of distribution is 7–9 l/kg. The average terminal half-life is 42 hours. The elimination is 59% via the gall bladder and feces and 33% via urine. The approved indications are complicated skin and soft tissue infections as well as complicated intra-abdominal infections.

### Ansamycins: rifampicin

In vitro, rifampicin works well against mycobacteria, staphylococci, including methicillin-resistant strains, streptococci and *Enterococcus* *faecalis* amongst others. Its effect on proliferating cells is highly bactericidal to bacteriostatic, depending on the dosage and activity of the pathogen. Because of the high likelihood of rapid development of resistance, rifampicin should not be given in monotherapy. Rifampicin is 70–90% protein bound. The substance penetrates the membrane well and accumulates intracellularly. The relative volume of distribution is >1 l/kg body weight. The half-life depends on the duration of treatment. In long-term treatment, autoinduction of metabolization will result in values of 2–3 hours. Rifampicin is eliminated via the gall bladder and kidneys. When rifampicin is used in patients undergoing renal replacement therapy, relevant drug adsorption at the filter must be expected. Whether and at which amount of the drug saturation of adsorption at the dialysis filter occurs has not been studied in detail. These relevant considerations should be taken into account when using rifampicin, especially in critically ill patients [9].

The most common adverse effects are liver function and gastrointestinal disorders. Blood count changes are possible. Rifampicin is a potent inducer of the cytochrome P450 enzyme system and therefore has a high potential for interaction.

### Nitroimidazoles: metronidazole

The antibacterial spectrum includes anaerobic Gram-positive and Gram-negative bacteria, with the exception of propionibacteria and actinomycetes. Metronidazole shows concentration-dependent bactericidal action. The relative volume of distribution is ca. 0.5 l/kg body weight, the half-life is 6–8 hours. Metronidazole is 10–20% bound to plasma proteins. It is metabolized and mainly excreted via the kidneys. Metronidazole is approved for the treatment of proven or suspected infections by anaerobes in different locations (including brain abscess) and perioperative prophylaxis. Metronidazole is commonly used in combination with other antibiotics for the treatment of mixed aerobic anaerobic infections or for monotherapy of *Clostridium* *difficile*-associated disease.

Adverse effects are rare cases of peripheral and central neuropathies.

### Phosphonic acids: fosfomycin

The antibacterial spectrum is broad and includes Gram-positive and Gram-negative pathogens, including MRSA, ESBL-producing Enterobacteriaceae and *Pseudomonas* *aeruginosa*. The mode of action is bactericidal. Fosfomycin is not bound to plasma proteins and is excreted unchanged via the kidneys. The average half-life is 2 hours. Penetration into different tissues is very good. Fosfomycin is approved for the treatment of many infections, including severe infections such as sepsis, meningitis, brain abscess, endocarditis, bone and joint infections, respiratory infections, skin/soft tissue infections, kidney and efferent urinary tract infections, and ear, nose and throat infections. Fosfomycin is not suitable for the monotherapy of severe infections. But it can be combined with a wide variety of antibiotics.

Most common undesirable effects are associated with high sodium content and increased potassium excretion.

### Inhibitors of folic acid synthesis: cotrimoxazole

Cotrimoxazole is the combination of sulfamethoxazole with trimethoprim. The antibacterial spectrum is broad and includes Gram-positive and Gram-negative pathogens, as well as some protozoa and *Pneumocystis* *jiroveci*. The distribution is extra and intracellular in both substances. The substances are metabolized in the liver. The half-life is 6.4 hours for active sulfamethoxazole and 7.8 hours for non-metabolized trimethoprim. Excretion is predominantly via the kidneys and partly via the liver and gall bladder. Cotrimoxazole, like many older antibiotics, is approved for a variety of indications. Reasonable indications include *Pneumocystis* pneumonia, infections caused by *Stenotrophomonas* *maltophilia* and nocardiosis. Especially with prolonged use reversible depressions of bone marrow or allergic reactions (up to Stevens-Johnson or Lyell syndrome) occur.

### Fusidic acid (parenteral formulation currently not available in Germany)

Fusidic acid has excellent activity against staphylococci but is inadequate against streptococci and has no activity against Gram-negative bacteria. In order to avoid development of resistance during prolonged therapy (e.g. osteomyelitis) combination with a second antibiotic effective against staphylococci is recommended. The current standard dosage is 500 mg orally or intravenously 3 to 4 times daily. There may be a transient increase in alkaline phosphatase. Parenteral administration must be carried out over at least four hours, since the substance is irritating to the venous wall. 

### Cyclic lipopeptides: daptomycin

Daptomycin is only effective against Gram-positive bacteria, including multi-drug resistant pathogens such as MRSA and VRE. The mode of action is bactericidal, both in the growth phase and in the stationary phase of the pathogens. The half-life is 8–9 hours, protein binding is 92%. The volume of distribution is given as 0.1 l/kg body weight. The substance is mainly eliminated via the kidneys; 5% is excreted via feces. Daptomycin is approved for the treatment of bacteremia, endocarditis and skin soft tissue infections [10], [11], [12]. It is not suitable for the treatment of pulmonary infections as daptomycin is inactivated by surfactant. 

### Polymyxins: colistin

Colistin acts exclusively on Gram-negative pathogens, including multidrug-resistant strains of *Pseudomonas* *aeruginosa*, *Acinetobacter* *baumannii* and ESBL- or carbapenemase-producing Enterobacteriaceae. *Proteus* spp., *Morganella* *morganii*, *Serratia* *marcescens*, *Burkholderia* *cepacia* complex, *Neisseria* spp. and *Moraxella* *catarrhalis* are resistant. The mode of action is bactericidal. Current data on pharmacokinetics and pharmacodynamics are now available on a larger scale, so the dosage regimes can be adjusted. Previously commonly reported side effects of nephrotoxicity and neurotoxicity are noted less frequently in recent case series and studies. Colistin in parenteral form is only suitable for the treatment of infections caused by multidrug-resistant Gram-negative pathogens [13].

## Note

This is the first chapter of the guideline “Calculated initial parenteral treatment of bacterial infections in adults – update 2018” in the 2^nd^ updated version. The German guideline by the Paul-Ehrlich-Gesellschaft für Chemotherapie e.V. (PEG) has been translated to address an international audience.

## Competing interests

The authors declare that they have no competing interests.

## Figures and Tables

**Table 1:**
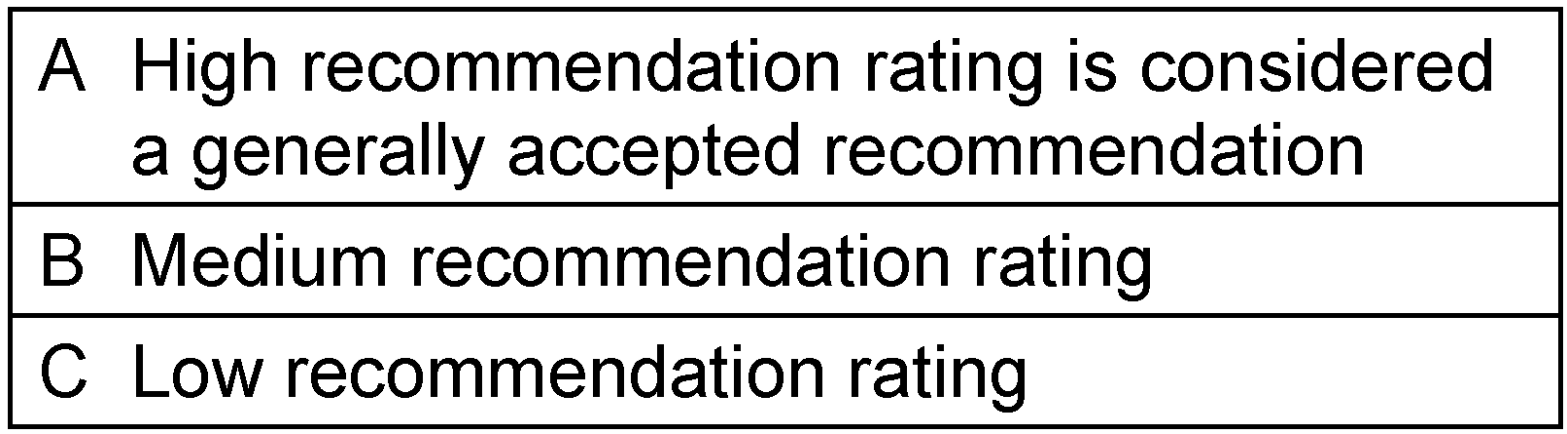
Recommendation ratings
